# Supplementary material for: Identification of the CNGC Gene Family in Rice and Mining of Alleles for Application in Rice Improvement
Source: Plants (Basel). 2023 Dec 6;12(24):4089. doi: 10.3390/plants12244089 (PMC10747058; doi:10.3390/plants12244089)
Supplement: Supplementary file 1 [file plants-12-04089-s001.zip › Supplementary Figure.pdf]

Identification of the CNGC gene family in rice and mining of alleles for application in rice improvement

Figure A1. The expression level of the *OsCNGC* genes in different rice tissues under the normal conditions. The plant tissues mainly include aleurone, anther,callus, leaf, panicle, pistil, root, seed and shoot.

Figure A2. Haplotype networks of the four cloned CNGC (1~4) genes and their association traits with four agronomic traits in 3KRG. p-values indicate differences among haplotypes assessed by two-factor ANOVA, where different letters on the box-and-line plots indicate statistically significant differences based on the Duncan's Multiple Range Test at P<0.05. The bars on the right show the frequency differences in dominant gcHaps between local varieties (LANs) and modern varieties (MVs) in Xi'an and Geng. The chi-square test was used to determine significant differences in the proportions of the same gcHap between groups ****P<0.0001, ***P<0.001, **P<0.01, *P<0.05 and N.S., not significant.

Figure A3. Haplotype networks of the four cloned CNGC(5~8) genes and their association traits with four agronomic traits in 3KRG. p-values indicate differences among haplotypes assessed by two-factor ANOVA, where different letters on the box-and-line plots indicate statistically significant differences based on the Duncan's Multiple Range Test at P<0.05. The bars on the right show the frequency differences in dominant gcHaps between local varieties (LANs) and modern varieties (MVs) in Xi'an and Geng. The chi-square test was used to determine significant differences in the proportions of the same gcHap between groups ****P<0.0001, ***P<0.001, **P<0.01, *P<0.05 and N.S., not significant.

Figure A4. Haplotype networks of the four cloned CNGC(10~12,15) genes and their association traits with four agronomic traits in 3KRG. p-values indicate differences among haplotypes assessed by two-factor ANOVA, where different letters on the box-and-line plots indicate statistically significant differences based on the Duncan's Multiple Range Test at P<0.05. The bars on the right show the frequency differences in dominant gcHaps between local varieties (LANs) and modern varieties (MVs) in Xi'an and Geng. The chi-square test was used to determine significant differences in the proportions of the same gcHap between groups ****P<0.0001, ***P<0.001, **P<0.01, *P<0.05 and N.S., not significant.

Figure A5. Comparison and analysis of 15 agronomic traits among the predominant gcHap, unfavorable gcHap, and major gcHaps of *OsCNGC1*

Figure A6. Comparison and analysis of 15 agronomic traits among the predominant gcHap, unfavorable gcHap, and major gcHaps of *OsCNGC2*

Figure A7. Comparison and analysis of 15 agronomic traits among the predominant gcHap, unfavorable gcHap, and major gcHaps of *OsCNGC3*

Figure A8. Comparison and analysis of 15 agronomic traits among the predominant gcHap, unfavorable gcHap, and major gcHaps of *OsCNGC4*

Figure A9. Comparison and analysis of 15 agronomic traits among the predominant gcHap, unfavorable gcHap, and major gcHaps of *OsCNGC5*

Figure A10. Comparison and analysis of 15 agronomic traits among the predominant gcHap, unfavorable gcHap, and major gcHaps of *OsCNGC6*

Figure A11. Comparison and analysis of 15 agronomic traits among the predominant gcHap, unfavorable gcHap, and major gcHaps of *OsCNGC7*

Figure A12. Comparison and analysis of 15 agronomic traits among the predominant gcHap, unfavorable gcHap, and major gcHaps of *OsCNGC8*

Figure A13. Comparison and analysis of 15 agronomic traits among the predominant gcHap, unfavorable gcHap, and major gcHaps of *OsCNGC9*

Figure A14. Comparison and analysis of 15 agronomic traits among the predominant gcHap, unfavorable gcHap, and major gcHaps of *OsCNGC10*

Figure A15. Comparison and analysis of 15 agronomic traits among the predominant gcHap, unfavorable gcHap, and major gcHaps of *OsCNGC11*

Figure A16. Comparison and analysis of 15 agronomic traits among the predominant gcHap, unfavorable gcHap, and major gcHaps of *OsCNGC12*

Figure A17. Comparison and analysis of 15 agronomic traits among the predominant gcHap, unfavorable gcHap, and major gcHaps of *OsCNGC13*

Figure A18. Comparison and analysis of 15 agronomic traits among the predominant gcHap, unfavorable gcHap, and major gcHaps of *OsCNGC14*

Figure A19. Comparison and analysis of 15 agronomic traits among the predominant gcHap, unfavorable gcHap, and major gcHaps of *OsCNGC15*

Figure A20. Comparison and analysis of 15 agronomic traits among the predominant gcHap, unfavorable gcHap, and major gcHaps of *OsCNGC16*

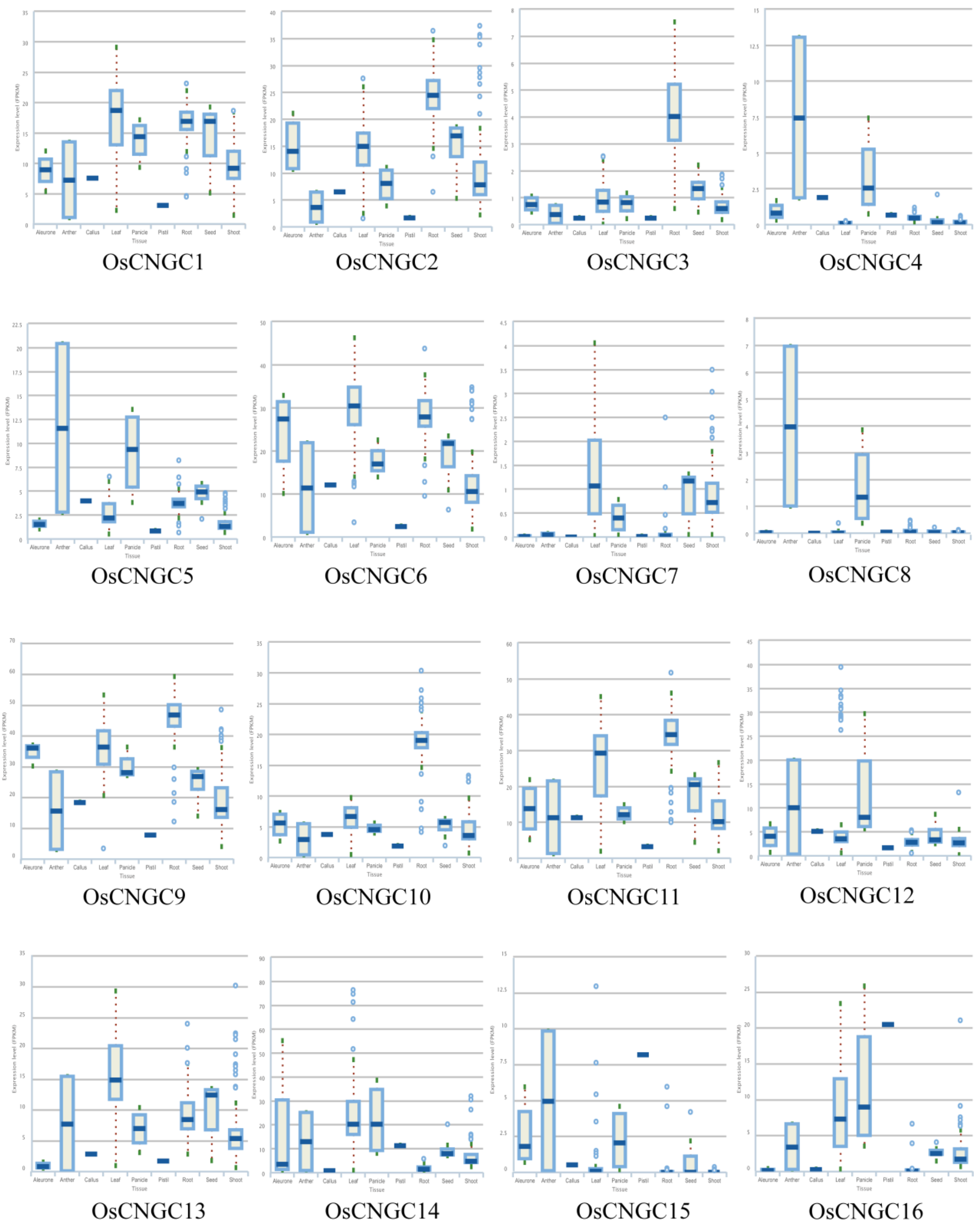

Figure A1. The expression level of the *OsCNGC* genes in different rice tissues under the normal conditions. The plant tissues mainly include aleurone, anther,callus, leaf, panicle, pistil, root, seed and shoot.

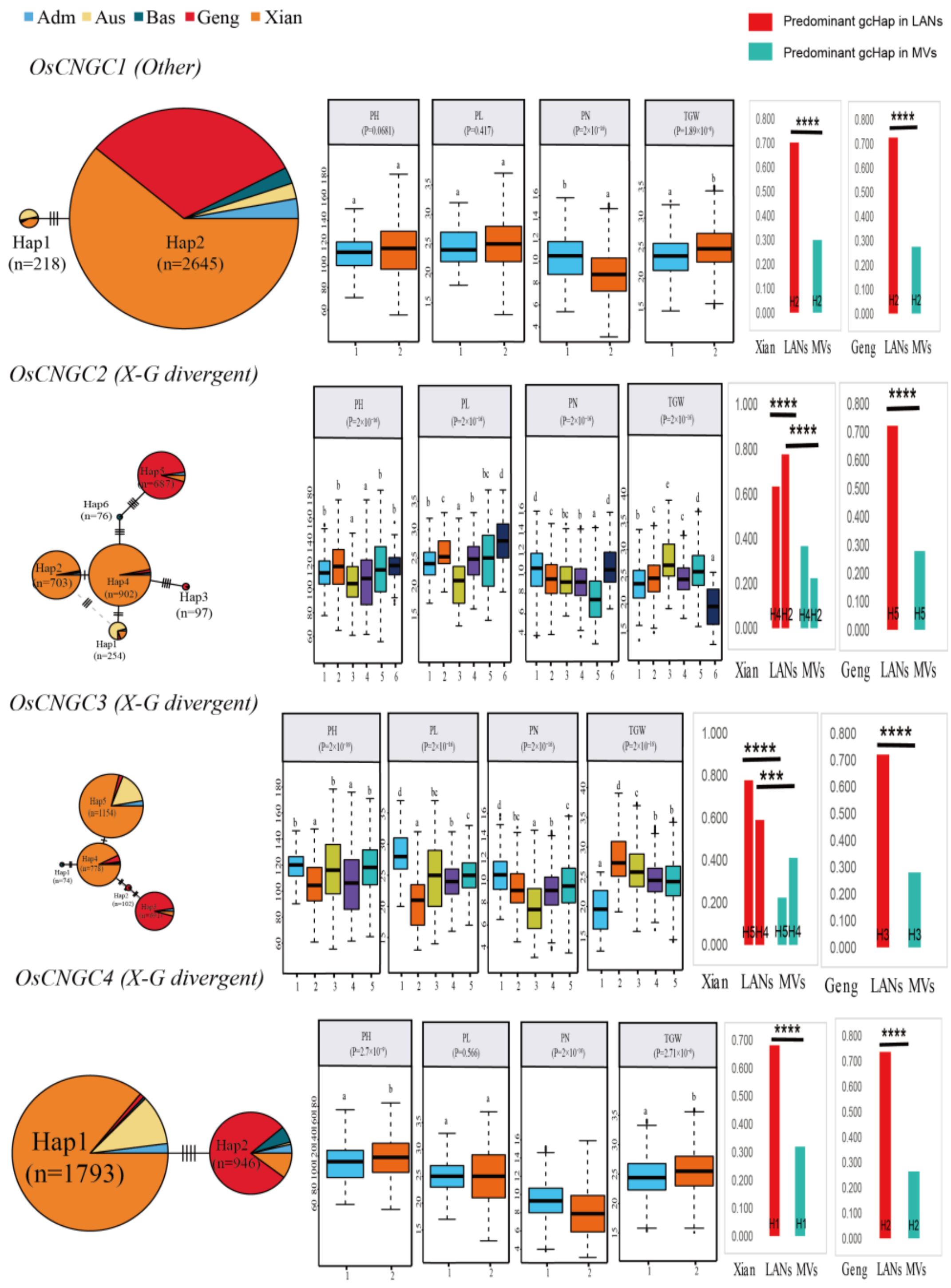

Figure A2. Haplotype networks of the four cloned *CNGC (1~4)* genes and their association traits with four agronomic traits in 3KRG. p-values indicate differences among haplotypes assessed by two-factor ANOVA, where different letters on the box-and-line plots indicate statistically significant differences based on the Duncan's Multiple Range Test at P<0.05. The bars on the right show the frequency differences in dominant gcHaps between local varieties (LANs) and modern varieties (MVs) in Xi'an and Geng. The chi-square test was used to determine significant differences in the proportions of the same gcHap between groups ****P<0.0001, ***P<0.001, **P<0.01, *P<0.05 and N.S., not significant.

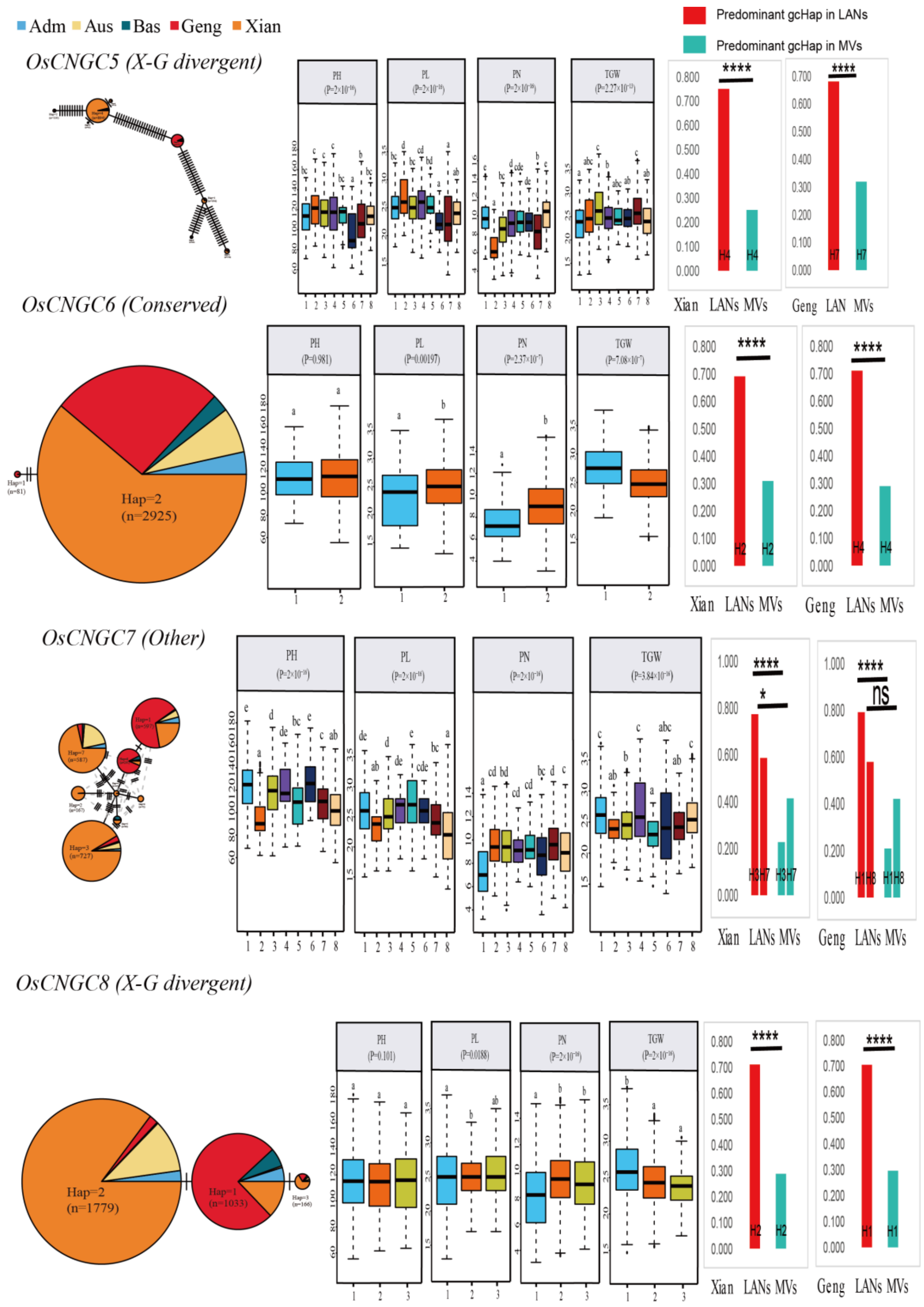

Figure A3. Haplotype networks of the four cloned *CNGC (5~8)* genes and their association traits with four agronomic traits in 3KRG. p-values indicate differences among haplotypes assessed by two-factor ANOVA, where different letters on the box-and-line plots indicate statistically significant differences based on the Duncan's Multiple Range Test at P<0.05. The bars on the right show the frequency differences in dominant gcHaps between local varieties (LANs) and modern varieties (MVs) in Xi'an and Geng. The chi-square test was used to determine significant differences in the proportions of the same gcHap between groups ****P<0.0001, ***P<0.001, **P<0.01, *P<0.05 and N.S., not significant.

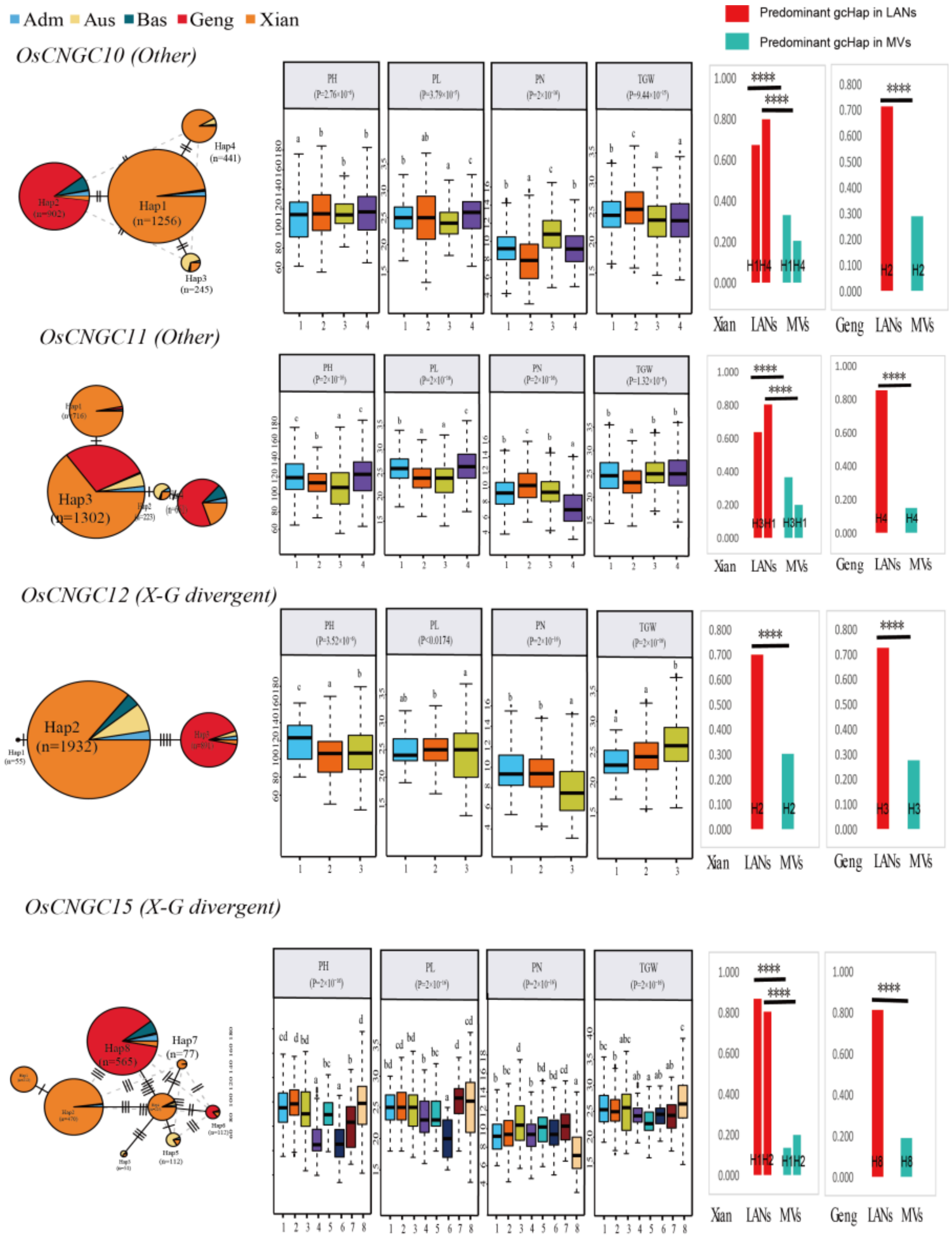

Figure A4. Haplotype networks of the four cloned *CNGC (10~12,15)* genes and their association traits with four agronomic traits in 3KRG. p-values indicate differences among haplotypes assessed by two-factor ANOVA, where different letters on the box-and-line plots indicate statistically significant differences based on the Duncan's Multiple Range Test at P<0.05. The bars on the right show the frequency differences in dominant gcHaps between local varieties (LANs) and modern varieties (MVs) in Xi'an and Geng. The chi-square test was used to determine significant differences in the proportions of the same gcHap between groups ****P<0.0001, ***P<0.001, **P<0.01, *P<0.05 and N.S., not significant.

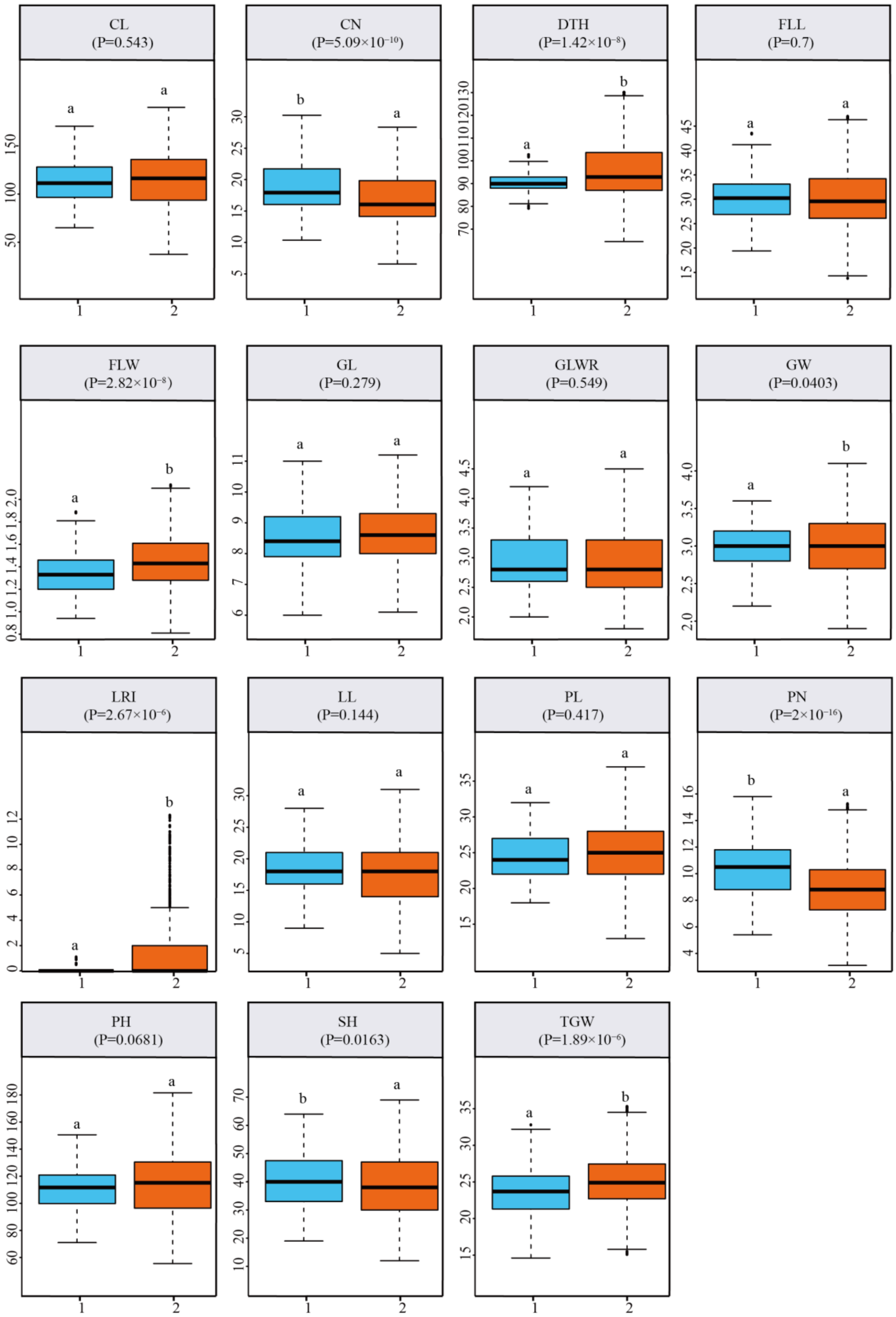

Figure A5. Comparison and analysis of 15 agronomic traits among the predominant gcHap, unfavorable gcHap, and major gcHaps of *OsCNGC1*

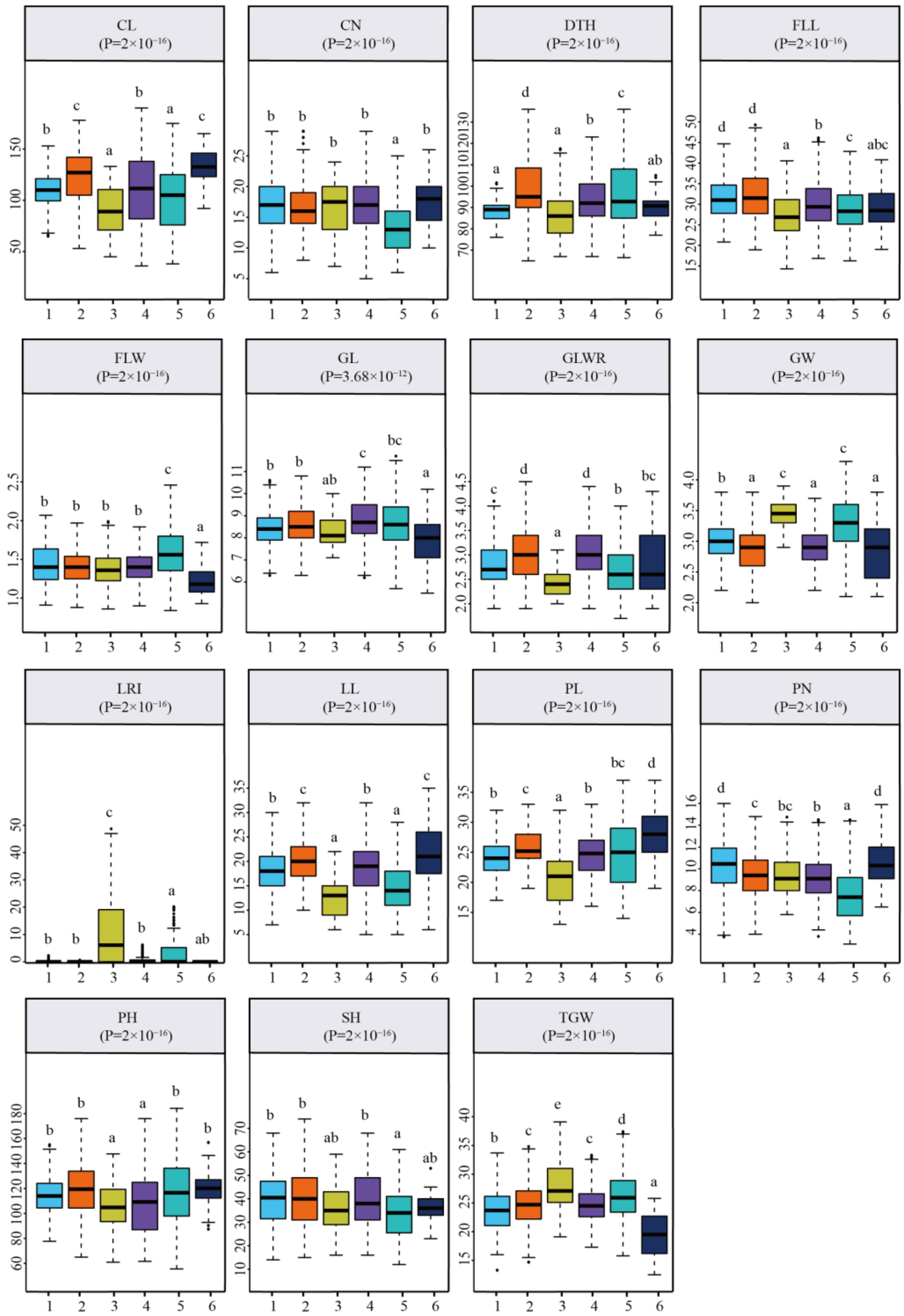

Figure A6. Comparison and analysis of 15 agronomic traits among the predominant gcHap, unfavorable gcHap, and major gcHaps of *OsCNGC2*

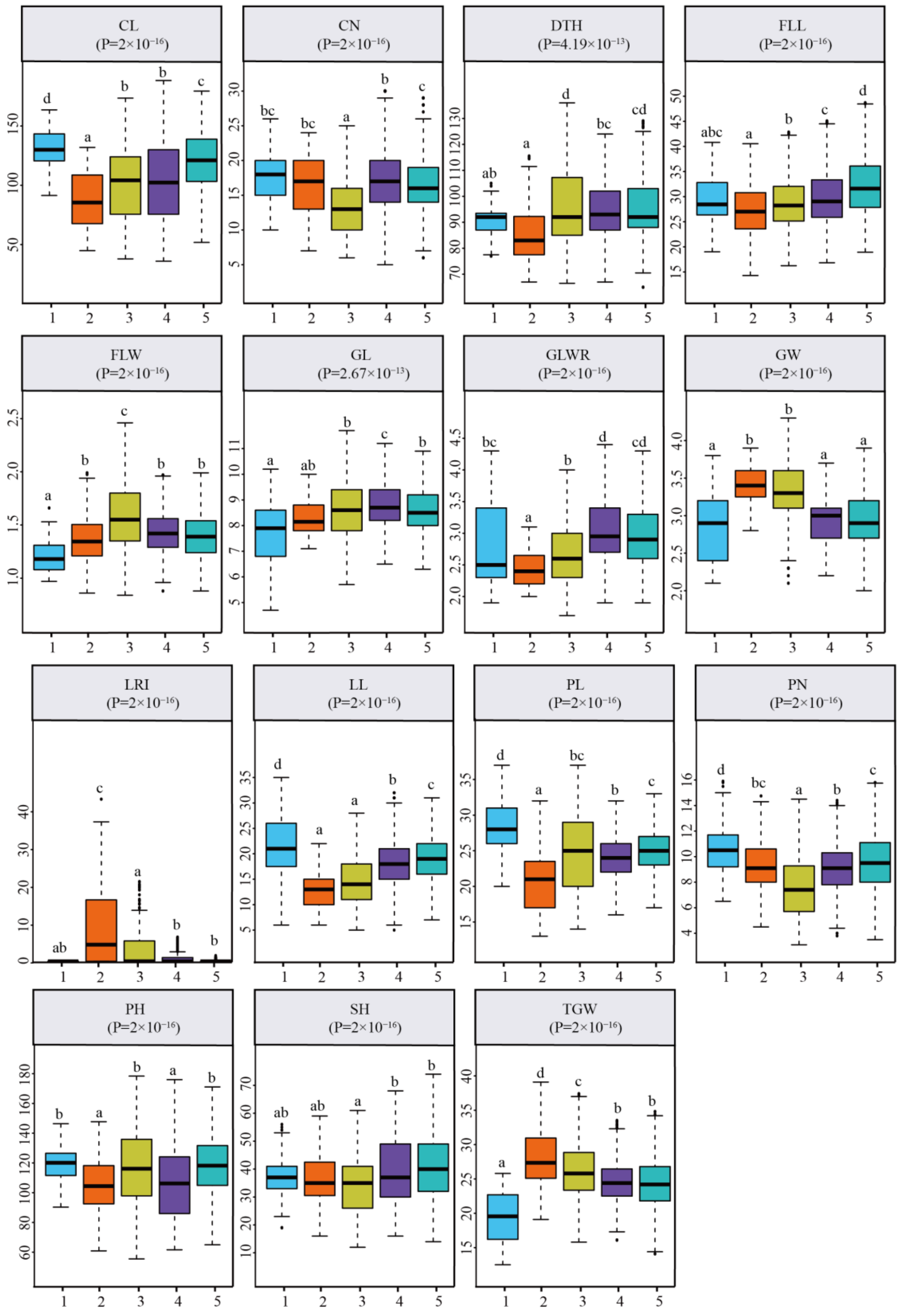

Figure A7. Comparison and analysis of 15 agronomic traits among the predominant gcHap, unfavorable gcHap, and major gcHaps of *OsCNGC3*

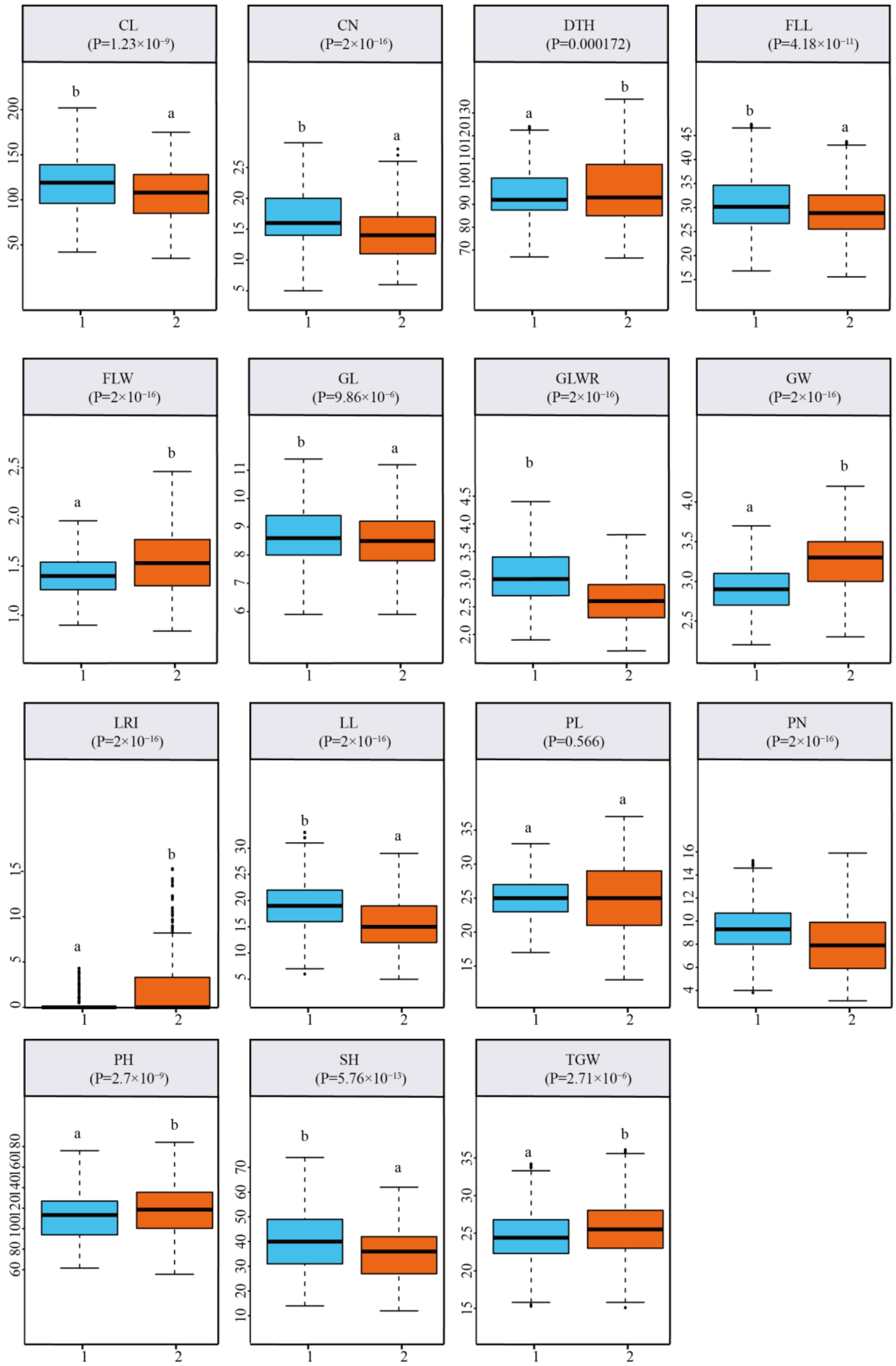

Figure A8. Comparison and analysis of 15 agronomic traits among the predominant gcHap, unfavorable gcHap, and major gcHaps of *OsCNGC4*

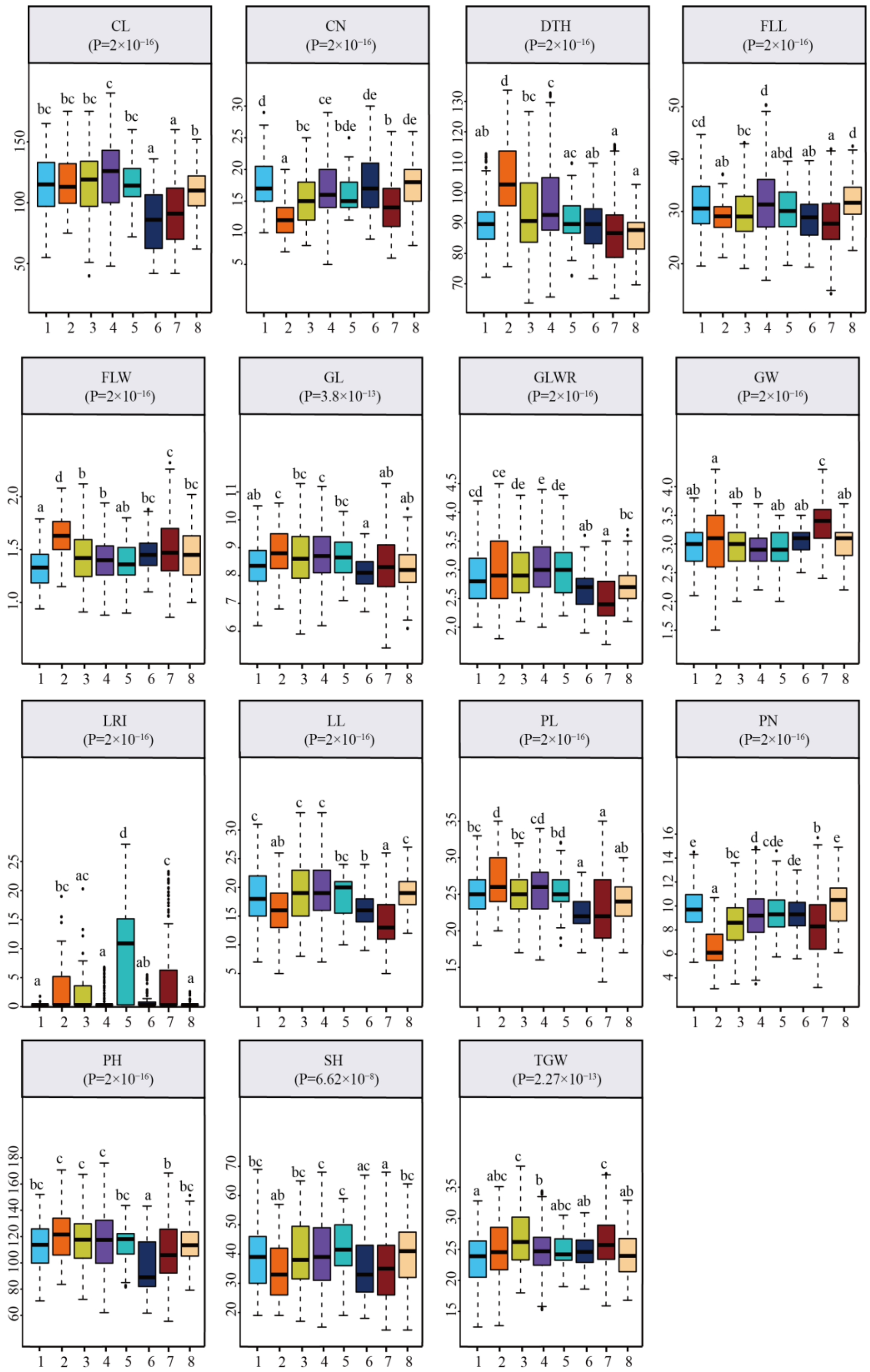

Figure A9. Comparison and analysis of 15 agronomic traits among the predominant gcHap, unfavorable gcHap, and major gcHaps of *OsCNGC5*

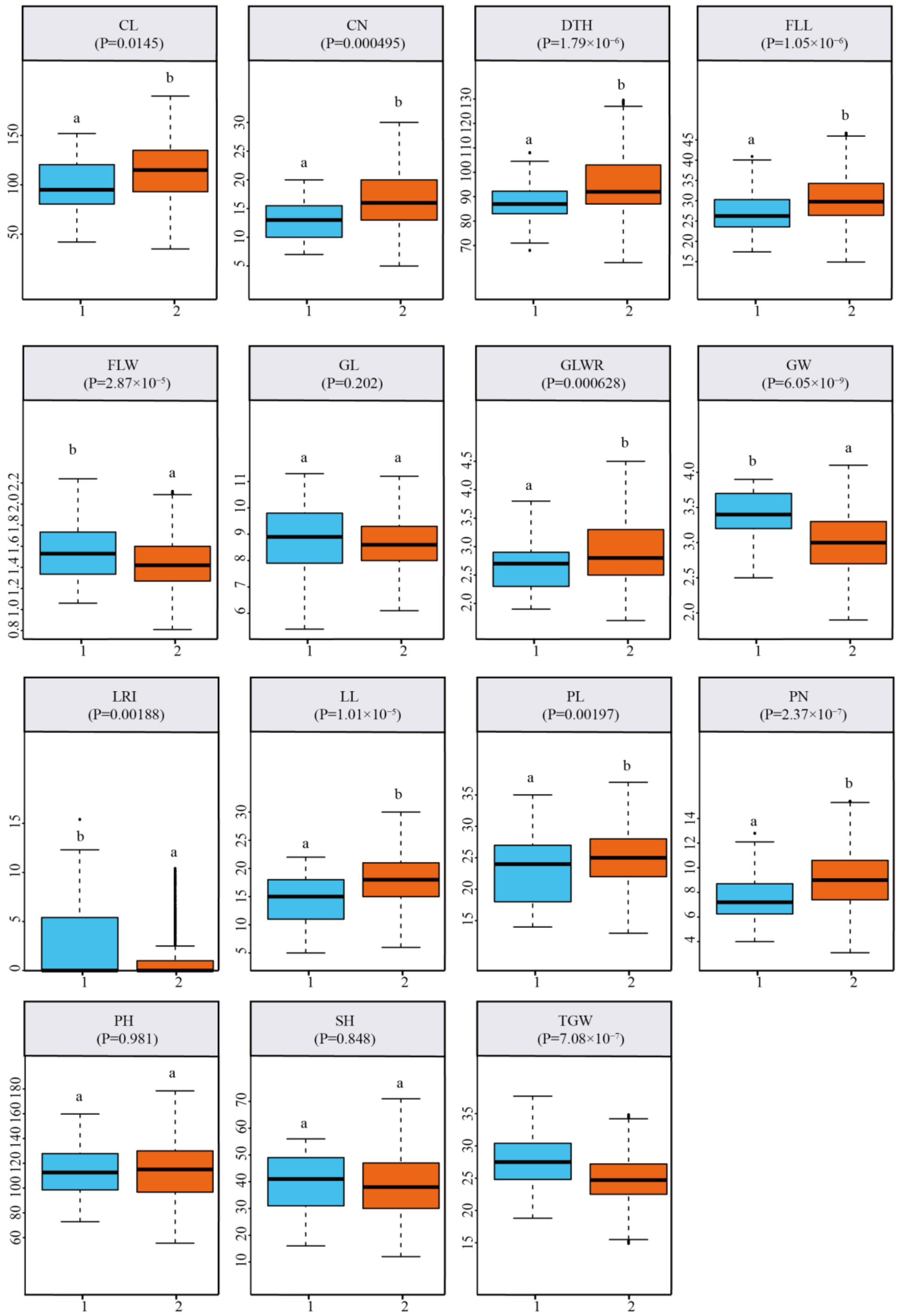

Figure A10. Comparison and analysis of 15 agronomic traits among the predominant gcHap, unfavorable gcHap, and major gcHaps of *OsCNGC6*

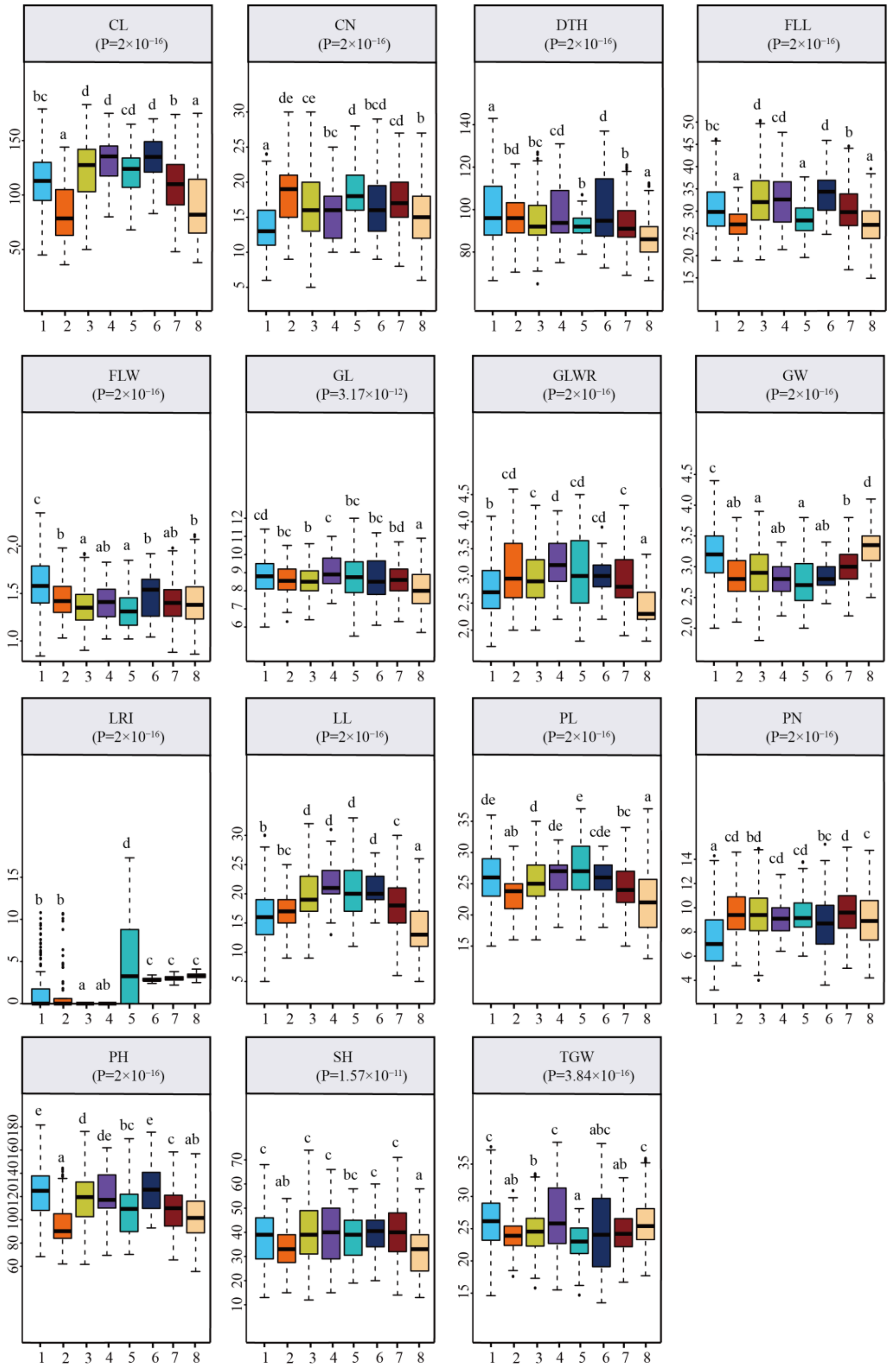

Figure A11. Comparison and analysis of 15 agronomic traits among the predominant gcHap, unfavorable gcHap, and major gcHaps of *OsCNGC7*

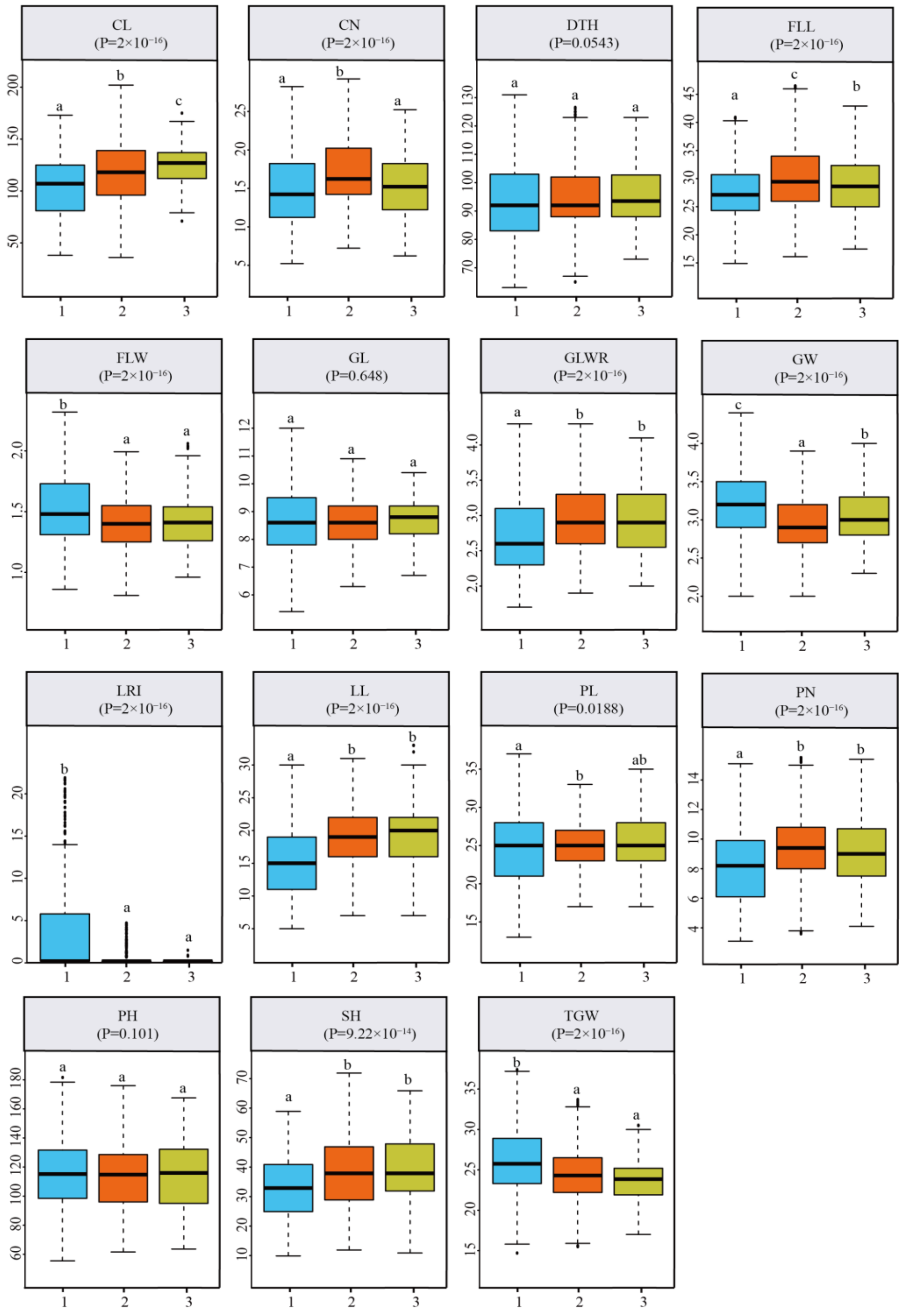

Figure A12. Comparison and analysis of 15 agronomic traits among the predominant gcHap, unfavorable gcHap, and major gcHaps of *OsCNGC8*

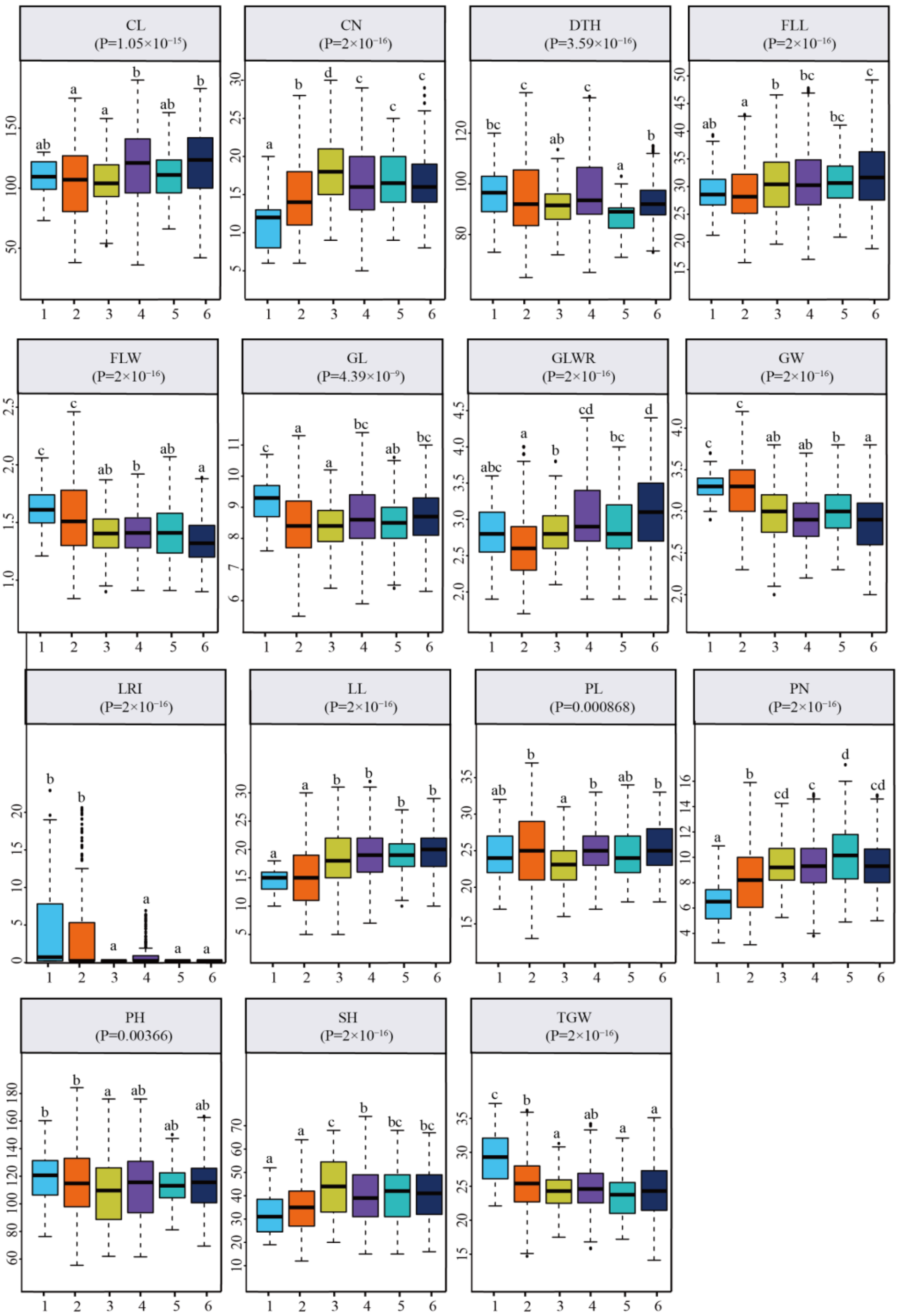

Figure A13. Comparison and analysis of 15 agronomic traits among the predominant gcHap, unfavorable gcHap, and major gcHaps of *OsCNGC9*

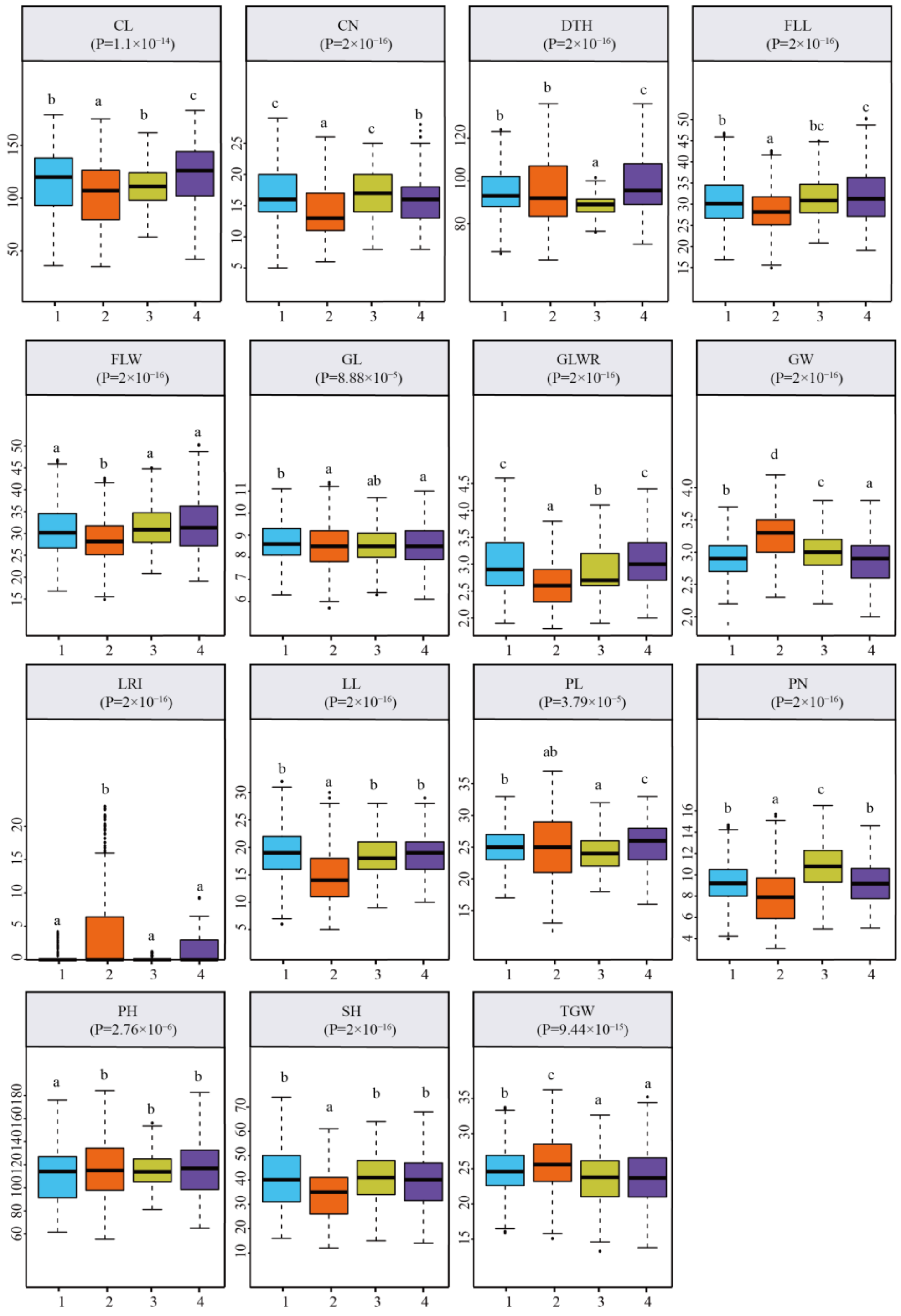

Figure A14. Comparison and analysis of 15 agronomic traits among the predominant gcHap, unfavorable gcHap, and major gcHaps of *OsCNGC10*

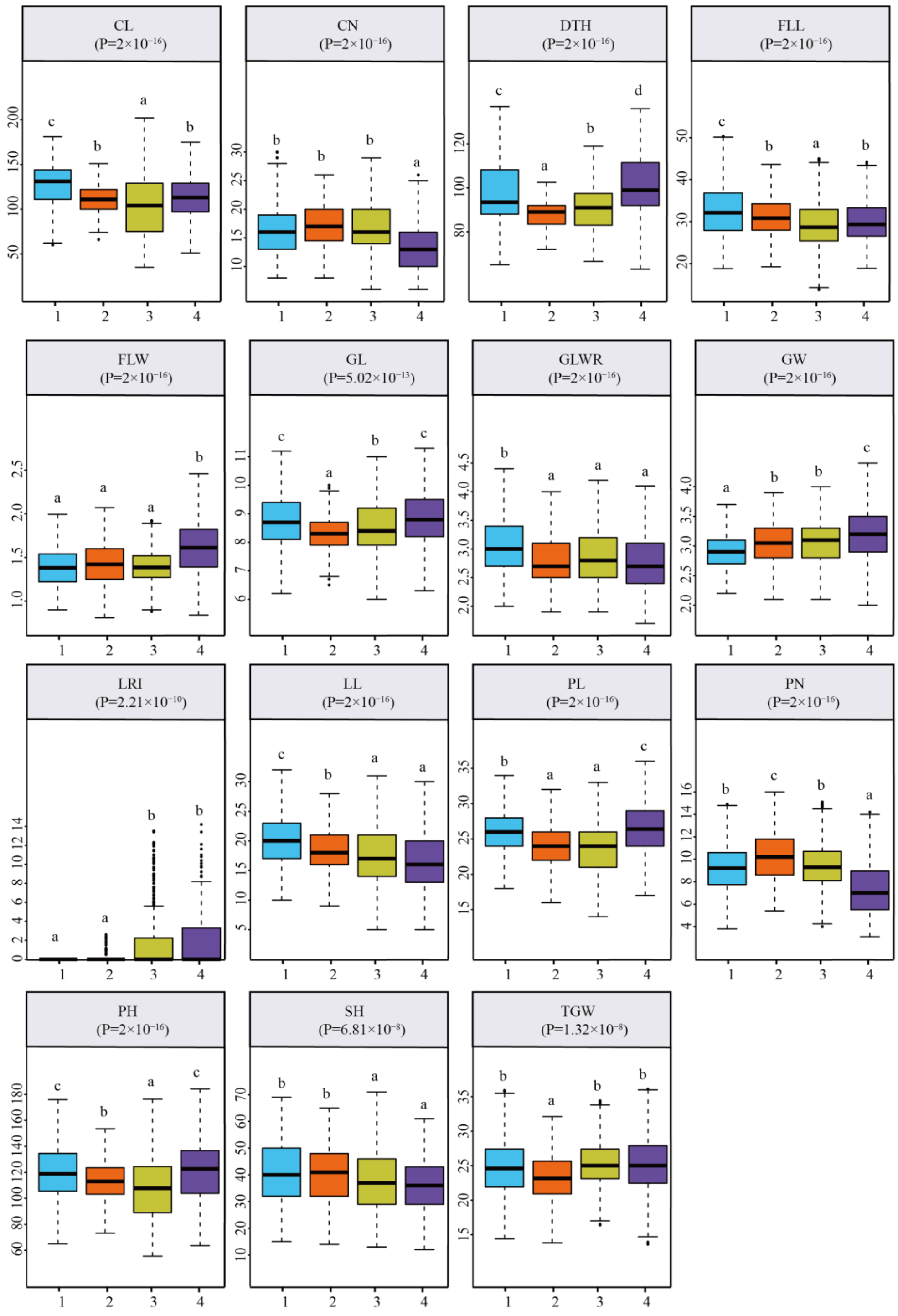

Figure A15. Comparison and analysis of 15 agronomic traits among the predominant gcHap, unfavorable gcHap, and major gcHaps of *OsCNGC11*

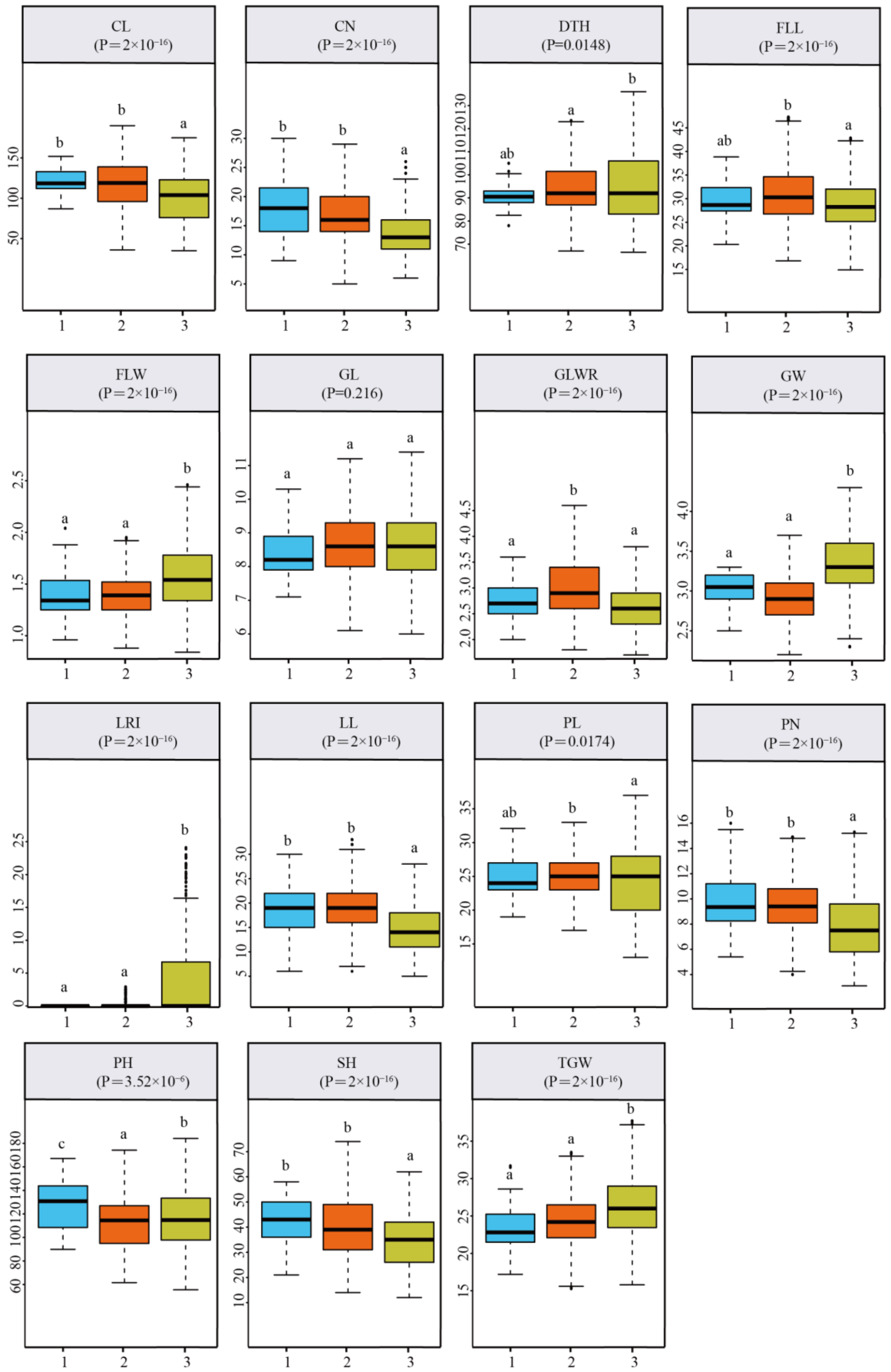

Figure A16. Comparison and analysis of 15 agronomic traits among the predominant gcHap, unfavorable gcHap, and major gcHaps of *OsCNGC12*

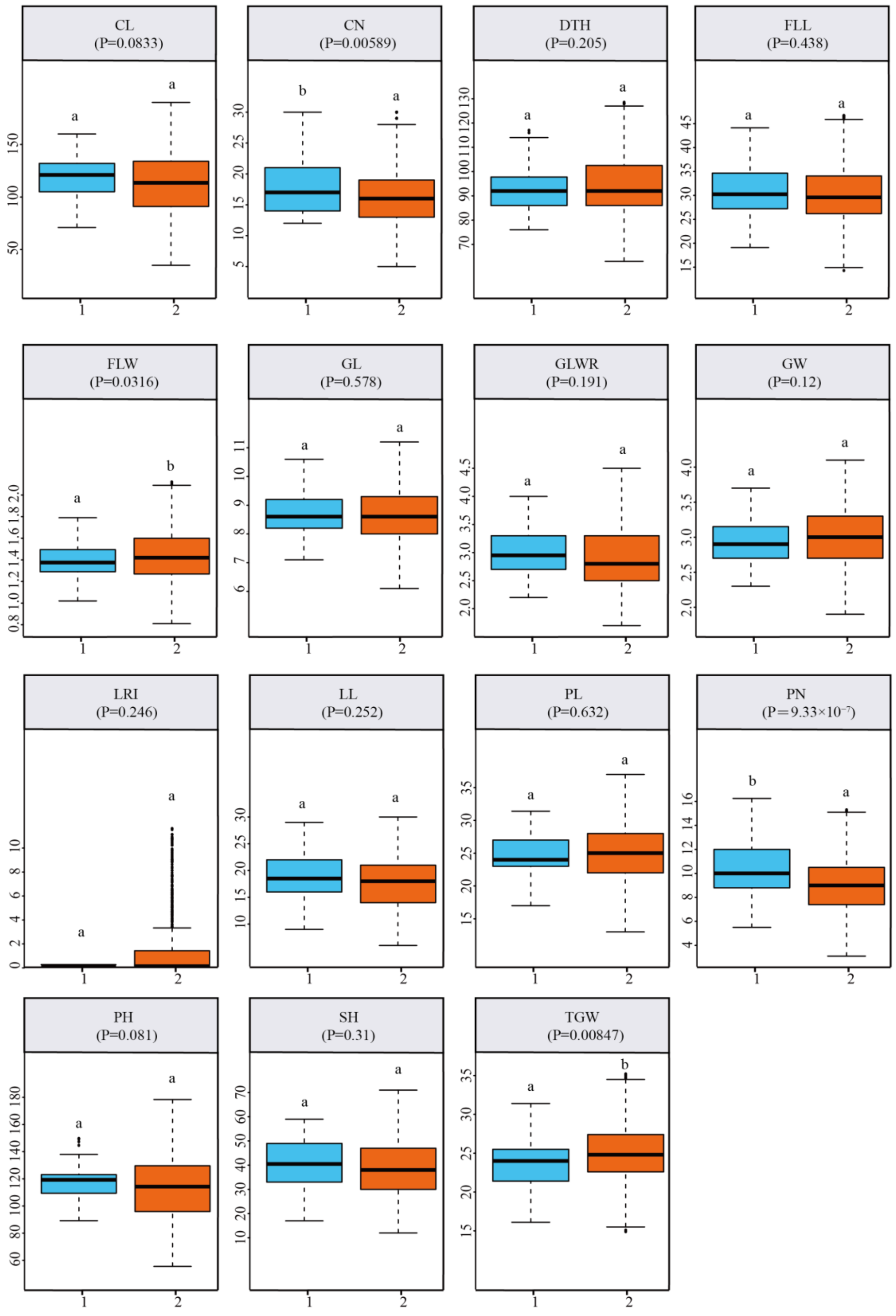

Figure A17. Comparison and analysis of 15 agronomic traits among the predominant gcHap, unfavorable gcHap, and major gcHaps of *OsCNGC13*

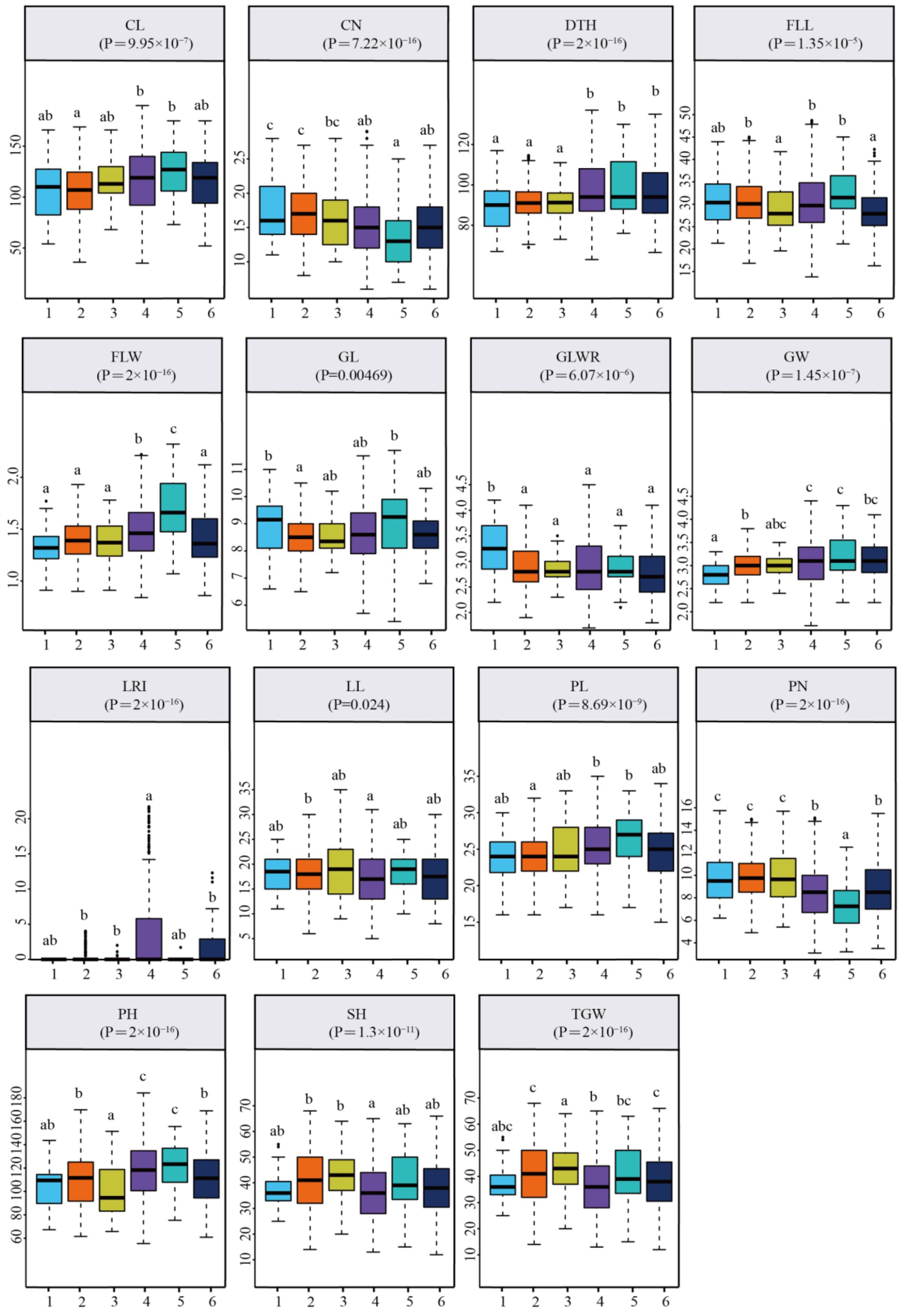

Figure A18. Comparison and analysis of 15 agronomic traits among the predominant gcHap, unfavorable gcHap, and major gcHaps of *OsCNGC14*

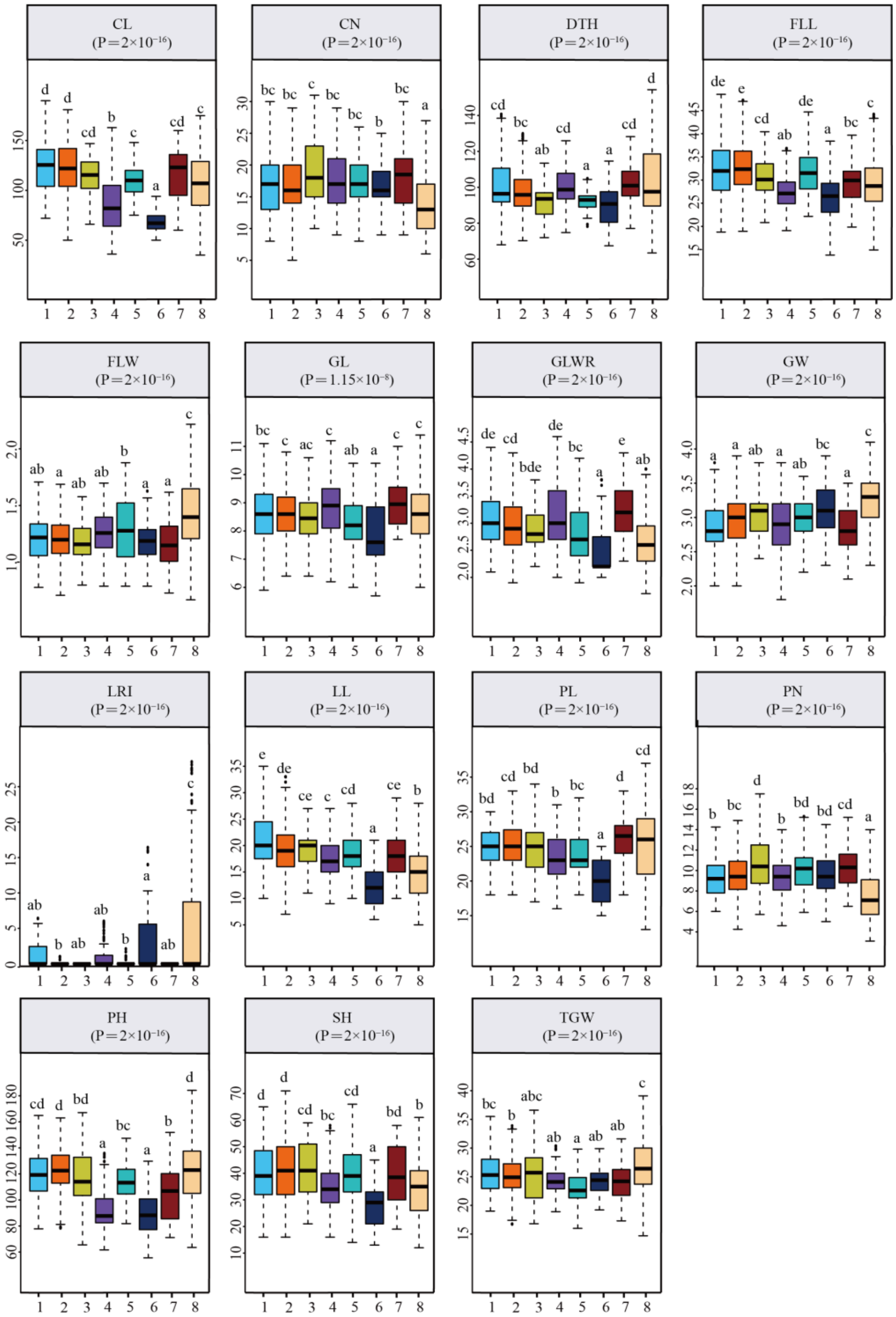

Figure A19. Comparison and analysis of 15 agronomic traits among the predominant gcHap, unfavorable gcHap, and major gcHaps of *OsCNGC15*

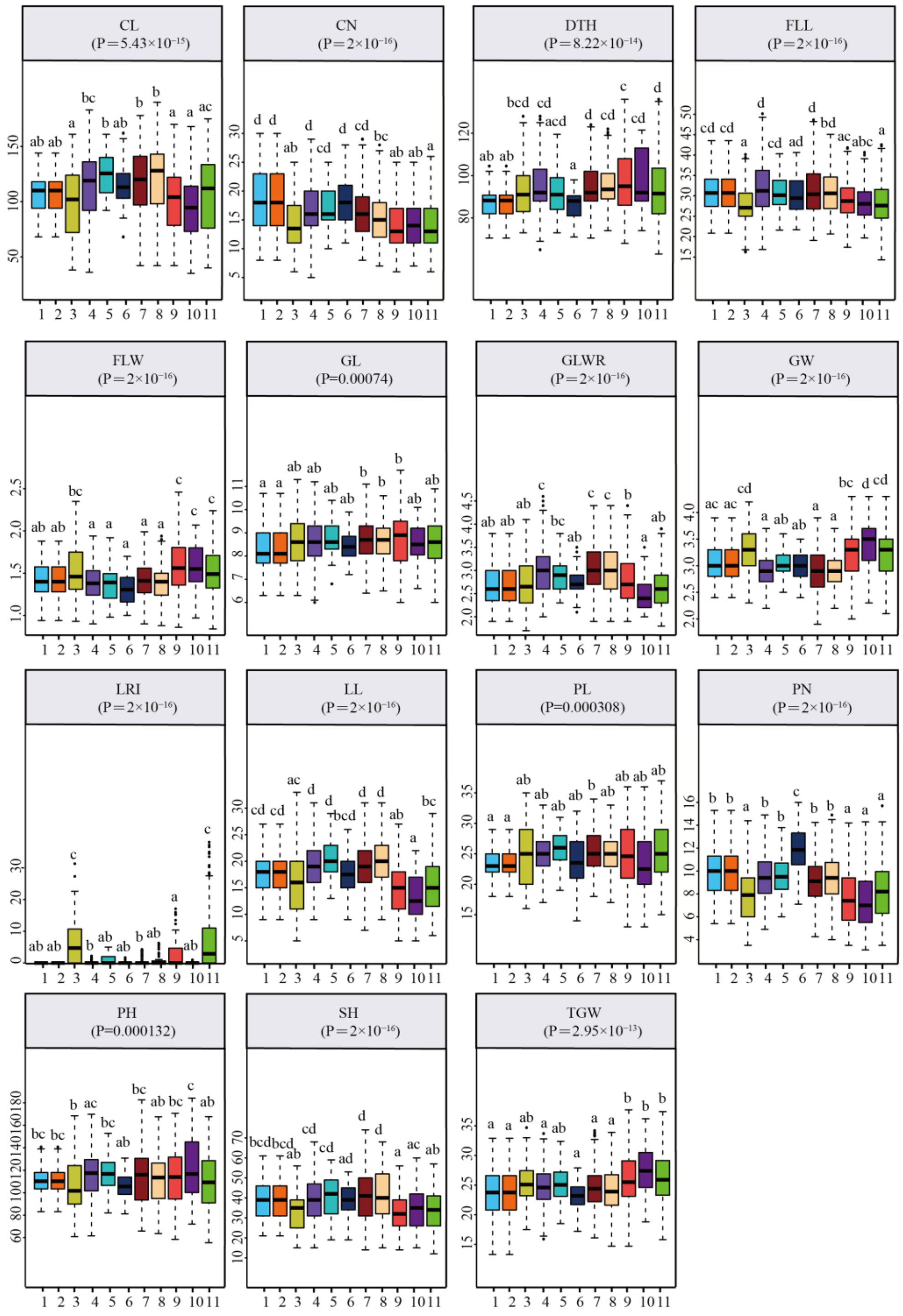

Figure A20. Comparison and analysis of 15 agronomic traits among the predominant gcHap, unfavorable gcHap, and major gcHaps of *OsCNGC16*
